# Clinical and Inflammatory Profile of COVID-19 Infection at a Tertiary Care Centre in Northern Part of Tamil Nadu – A Retrospective Study

**DOI:** 10.7759/cureus.30139

**Published:** 2022-10-10

**Authors:** Bhaskaran Shanmukham, Appandraj Srivijayan, Sivagamasundari Venugopal, Shyamala Ravikoti, Ariyanachi Kaliappan, Archana Gaur, Jeganathan Geetha, Varatharajan Sakthivadivel, Yuvaraj Balan, Raja Sundaramurthy

**Affiliations:** 1 General Medicine, Melmaruvathur Adhiparasakthi Institute of Medical Sciences and Research, Melmaruvathur, IND; 2 Paediatrics, Melmaruvathur Adhiparasakthi Institute of Medical Sciences and Research, Melmaruvathur, IND; 3 Microbiology, All India Institute of Medical Sciences, Bibinagar, IND; 4 Anatomy, All India Institute of Medical Sciences, Bibinagar, IND; 5 Physiology, All India Institute of Medical Sciences, Bibinagar, IND; 6 General Medicine, Karpaga Vinayaga Institute of Medical Sciences & Research Center, Maduranthagam, IND; 7 General Medicine, All India Institute of Medical Sciences, Bibinagar, IND; 8 Biochemistry, All India Institute of Medical Sciences, Bibinagar, IND

**Keywords:** intensive care unit, inflammatory profile, clinical profile, outcome, covid-19

## Abstract

Introduction

The coronavirus disease (COVID-19) pandemic has incurred high costs for the entire planet. The complex interactions between the host, virus, and environment have resulted in various clinical outcomes. It is crucial to comprehend sickness severity and outcome predictors to provide early preventative measures for a better outcome. The current study aimed to determine the association of clinical and inflammatory profiles with the outcome of COVID-19 infection in patients admitted to the intensive care unit.

Methods

This retrospective study was done in patients admitted to intensive care units for COVID-19 with a positive reverse transcriptase polymerase chain reaction (RTPCR) assay. A total of 125 patients above 18 years were included in the study. The patient’s age, gender, and co-morbidities like type 2 diabetes mellitus, hypertension, respiratory illness, and coronary artery disease were noted. The patient’s symptomatology, vital signs, oxygen saturation (Spo2), need for inotropes, and non-invasive positive pressure ventilator support (NIPPV) were observed. Computed tomography severity score (CTSS) and hematological and inflammatory parameters at the time of admission were noticed. Patient’s management and treatment outcomes as survivors and non-survivors were noted.

Results

The mean age was significantly greater in non-survivors. The common symptoms were fever, respiratory distress, cough, muscle pain, and sore throat. The leucocyte count, C-reactive protein (CRP), urea, creatinine, interleukin-6 (IL-6), and lactate dehydrogenase (LDH) were greater, and platelet counts were lower significantly in the non-survivors group. On multivariable logistic regression, CT severity score, NIPPV, and IL-6 had an odds ratio of 1.17, 0.052, and 1.03, respectively. IL-6 had a sensitivity of 81.5% and a specificity of 81.8% with a cut-off value of 37.5.

Conclusion

Vigilant monitoring of leucocyte count, CRP, urea, creatinine, IL-6, LDH, platelet count, and CT severity score is essential for managing COVID-19 infection. IL-6 was found to be a significant marker as a predictor of outcome in our study.

## Introduction

The COVID-19 pandemic has cost the entire globe a great deal. It is caused by a single-stranded RNA betacoronavirus called severe acute respiratory syndrome coronavirus 2 (SARS-CoV-2). It has been determined that angiotensin-converting enzyme 2 (ACE2) is the essential host receptor for SARS-CoV-2. ACE 2 is highly expressed in a broad range of cells found in several different human organs. The binding of the spike protein of the virus to the ACE 2 receptors of the host cell will cause the fusion of their membranes, which activates infection by releasing viral RNA into the cytoplasm. The initial signs vary from a low-grade fever, runny nose, and cough to shortness of breath and coma. Typically, 10-20% of patients develop a severe form of the condition that needs hospitalization or perhaps intensive care unit therapy. The complex interactions between the host, virus, and environment have resulted in various clinical outcomes [1].

Understanding illness severity and outcome predictors are essential to offer early preventative interventions for a better result. Studies have shown that advanced age, male sex, tobacco smoking, co-morbidities, particularly chronic kidney, respiratory, cardio-cerebrovascular diseases, and corticosteroid therapy were significant risk factors linked to an increased mortality rate with COVID-19 infection [2]. Identifying crucial laboratory indicators for disease severity at an early stage might assist in monitoring and preventing disease development towards a severe form in the lack of a targeted medication for the illness. Studies have shown that many laboratory indicators, including the total leucocyte count, C-reactive protein (CRP), urea, creatinine, interleukin-6 (IL-6), platelet count, D-dimer, ferritin, random blood sugar levels, and CT-severity score are connected to illness prognosis and outcome [3,4]. The current study was carried out to determine the association of clinical and inflammatory profiles with the outcome of COVID-19 infection. 

## Materials and methods

The study was conducted after obtaining Institute Ethics committee (Human Studies) permission [Melamaruvathur Adhiparasakthi Institute of Medical Sciences and Research (MAPIMS)/IEC/52/2022/210(5)2022] at a medical college hospital situated in the Northern District of Tamilnadu. This retrospective observational study was carried out on patients admitted to the intensive care unit with COVID-19 infection. The data was obtained from the case records at the hospital's medical records department for the period of July 2021 to August 2021. Patient demographic data like age, gender, and co-morbidities like diabetes mellitus, hypertension, respiratory illness, and coronary artery diseases were observed. The patient's symptoms, vitals, oxygen saturation (Spo2), need for inotropes, and non-invasive positive pressure ventilator support (NIPPV) were observed. CT severity indexes and hematological and inflammatory parameters at the time of admission were noticed. Patient's management and treatment outcomes as survivors and non-survivors were noted.

Statistical analysis

The data were entered in Microsoft Excel and analyzed using SPSS software version 25 (IBM, USA). Continuous variables were represented as mean±SD, and categorical variables were represented as frequency and percentage. The means of a selected quantitative variable of survivors and non-survivors were compared using an independent t-test. For the comparison of categorical variables, the chi-square test was used. Multivariable logistic regression was done to assess the predictors of mortality. Receiver-operating characteristic (ROC) was done to find the cut-off value of IL-6 to predict the outcome in COVID-19 patients. A p-value of 0.05 was considered statistically significant.

## Results

A total of 160 patients data were collected. Thirty-five patients were not included because of insufficient data. The non-survivors outnumbered the survivors in our study. The mean age of non-survivors was significantly greater than survivors, and the gender did not significantly differ between survivors and non-survivors. Hypertension as a co-morbidity was significantly more in the non-survivor group. The presence of other co-morbidities like diabetes mellitus, respiratory diseases, and coronary artery disease was similar in both groups except for hypothyroidism which was greater in the survivor group, though not significant (Table 1).

**Table 1:** Basic characteristics of patients with COVID-19 infection *Signifies P value <0.05

Parameter	Survivors (n=44)	Non-Survivors (n=81)	P value
Age (Years)	49.91 ± 15.10	61.77 ± 13.89	0.000*
Male	32 (72.7)	60 (74.1)	1.000
Female	12 (27.3)	21 (25.9)	
Co-morbidities			
Diabetes Mellitus	19 (43.2)	41 (50.6)	0.458
Hypertension	10 (22.7)	39 (48.1)	0.007*
Respiratory diseases	2 (4.5)	4 (4.9)	1.000
Hypothyroidism	4 (9.1)	1 (1.2)	0.052
Coronary artery disease	6 (13.6)	11 (13.6)	1.000
Cancer	1 (2.3)	-	0.303
Immunosuppression	-	2 (2.5)	0.540
Vaccination	-	2 (2.5)	0.540

The common symptoms in our study were fever, respiratory distress, cough, muscle pain, sore throat, and diarrhea, which did not differ among the groups. The mean heart rate, blood pressure, requirement for invasive ventilation, and hemodialysis did not differ significantly in both groups. Also, the requirement for drugs like inotropes, anti-viral drugs, and duration of stay was similar in both groups. However, the Spo2 was significantly lower in the non-survivors group, and the CT severity score and NIPPV were significantly more significant in the non-survivors group (Table 2).

**Table 2:** Clinical profile of the study population NIPPV- non-invasive positive pressure ventilator *Signifies P value <0.05, Spo2 – Oxygen saturation, CT – Computed tomography

Parameter	Survivors (n=44)	Non-Survivors (n=81)	P value
Fever	34 (77.3)	66 (81.5)	0.642
Cough	31 (70.5)	56 (69.1)	0.746
Respiratory distress	31 (70.5)	66 (81.5)	0.181
Sore throat	9(20.5)	25 (30.9)	0.293
Muscle pain	17(38.6)	41 (50.6)	0.586
Diarrhea	3 (6.8)	10 (12.3)	0.541
Heart rate	90.16 (17.9)	94.8 (15.8)	0.154
Systolic blood pressure	126.02 (15.8)	126.79 (15.7)	0.797
Diastolic blood pressure	77.36 (9.2)	78.67 (9.4)	0.459
SpO_2_	95.25 (3.9)	91.88 (6.1)	0.000*
CT severity score	12.98 (7.48)	21.31 (9.82)	0.000*
NIPPV	3 (6.8)	56 (69.1)	0.000*
Invasive ventilation	1 (2.3)	3 (3.7)	1.000
Inotropes	1 (2.3)	1 (1.2)	1.000
Hemodialysis	1 (2.3)	2 (2.5)	0.758
Remidesivir	23 (52.3)	42 (51.9)	1.000
Bevacizumab	4 (9.1)	7 (8.6)	1.000
Tocilizumab	1 (2.3)	1 (1.2)	1.000
Dexamethasone	40 (88.8)	81 (100)	1.000
Duration of stay in days	6.3 (3.56)	5.59 (2.88)	0.265

The total leucocyte count, CRP, urea, creatinine, IL-6, and lactate dehydrogenase (LDH) were significantly greater, and platelet counts were significantly lower in the non-survivors group. The random blood sugar, D-Dimer, and Ferritin were also higher in the non-survivors group but insignificant (Table 3).

**Table 3:** Inflammatory profile and biochemical parameters among survivors and Non-Survivors *Signifies P value <0.05

Parameter	Survivor (n=44)	Non-Survivor (n=81)	P value
Hemoglobin (gms/dl)	12.57 ± 2.18	13.05 ± 2.37	0.257
Total leucocyte count (/μL)	8108.82 ± 3587.41	10861.81 ± 4958.6	0.001*
Platelet count (x 10^3^)	250.79 ± 121.21	197.43 ± 76.12	0.010*
CRP (mg/dl)	50.57 ± 61.98	82.68 ± 64.18	0.008*
Total protein (gms/dl)	6.75 ± 0.91	6.53 ± 0.79	0.185
Albumin (gms/dl)	3.77 ± 0.91	3.65 ± 0.55	0.284
Aspartate Transaminase (U/L)	56.29 ± 56.24	48.99 ± 21.45	0.482
Alanine Transaminase (U/L)	38.43 ± 23.88	42.04 ± 35.39	0.500
Random blood sugar (mg/dl)	198.64 ± 107.65	225.37 ± 110.44	0.192
Urea (mg/dl)	34.61 ± 20.75	50.89 ± 36.16	0.002*
Creatinine (mg/dl)	1.09 ± 0.47	1.44 ± 1.32	0.033*
Interleukin-6 (pg/ml)	25.77 ± 23.13	181.49 ± 218.63	0.000*
D-dimer (μg/ml)	0.98 ± 2.05	1.96 ± 6.64	0.221
Lactate Dehydrogenase (U/L)	607.41 ± 323.06	757.28 ± 480.93	0.040*
Ferritin (ng/ml)	641.23 ± 517.48	676.15 ± 480.61	0.713

The multivariable logistic regression for predictors of mortality showed CT severity score, NIPPV, and IL-6 with a relative risk of 1.17, 0.052, and 1.03, respectively. The total count had a relative risk of 1 (Table 4).

**Table 4:** Multivariable logistic regression for predictors of mortality in patients with COVID-19 infection NIPPV – Non-invasive positive pressure ventilation, CT – Computed tomography

Parameter	RR (95% CI)	P value
CT severity score	1.17 (1.05-1.31)	0.005
NIPPV	0.052 (0.008-0.33)	0.002
Interleukin-6	1.03 (1.01-1.05)	0.004
Total count	1 (1-1)	0.012

**Figure 1:**
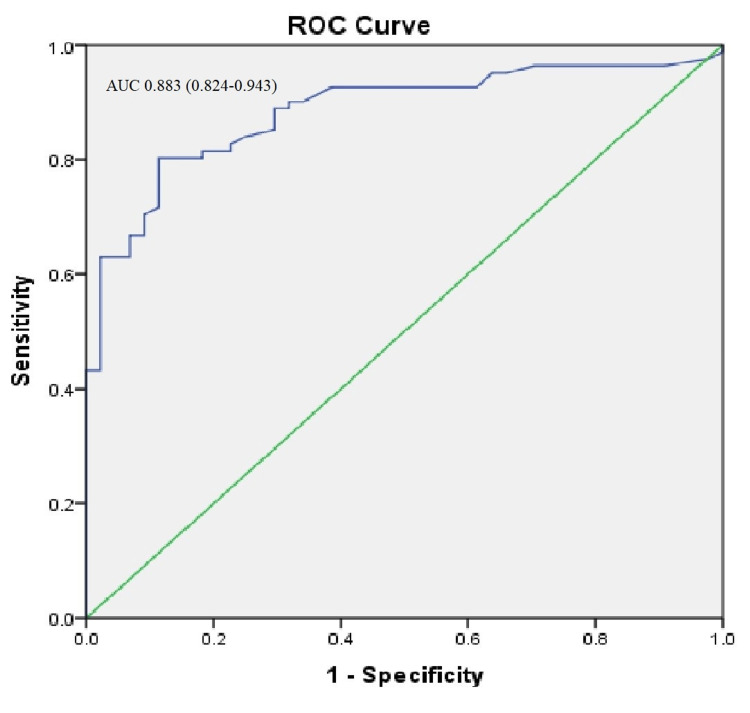
ROC curve of Interleukin-6 as predictors of COVID-19 outcome

The inflammatory marker IL-6 proved to be a better indicator of the outcome of COVID-19 infection, with a sensitivity of 81.5% and specificity of 81.8% with a cut-off value of 37.5 (Table 5 & Figure 1).

**Table 5:** The performance criteria of the Interleukin-6 in discriminating the outcome of COVID-19 infection AUC – Area under curve, CI – confidence interval

Parameter	AUC (95%CI)	Sensitivity	Specificity	Cut of Value	P value
Interleukin-6	0.883 (0.824-0.943)	81.5	81.8	37.5	0.000

## Discussion

In our study, the non-survivors outnumbered the survivors. This was due to more elderly individuals and co-morbidities, especially hypertension in the non-survivors group. The non-survivors were significantly older (61.77 ± 13.89 years) than the survivors (49.91 ± 15.10 years). This is consistent with the previous studies documenting the age-related increase in COVID-19 mortality [4,5]. This could be attributed to natural immunity steadily weakening with age, making older persons more prone to developing more severe illnesses [6]. Another study hypothesized that patients over 50 may have greater levels of ACE2, encoded by the ACE2 gene, and may be responsible for significantly higher risk of death [7]. The present study observed associated co-morbidities like hypertension, diabetes, coronary artery disease, and hypothyroidism among survivors and non-survivors. However, in this study, hypertension was significantly greater among the non-survivors. This is in line with similar other studies where significant correlations were found between hypertension and mortality of the COVID-19 disease [8,9].

In the present study, the common symptoms were fever, respiratory distress, cough, muscle pain, sore throat, and diarrhea, which did not differ among the groups. The mean heart rate, blood pressure, requirement for invasive ventilation, and hemodialysis did not differ significantly in both groups. Also, the requirement for drugs like inotropes, anti-viral drugs, and duration of stay was similar in both groups. However, the Spo2 was significantly lower (<91.88%) in the non-survivors group; the CT severity score and NIPPV were significantly greater in the non-survivors group. In a study done by Mejia et al., it was reported that the degree of hypoxemia (mostly Spo2 <85%) was independently related to in-hospital mortality [10]. Xie et al. also documented that for each 1-Unit increase in Spo2, mortality risk decreased by approximately 8% (HR, 0.92; 95% CI, 0.91 to 0.94; P<.001) [11]. In our study, the platelet count was significantly lower in non-survivors. In a study by Rampotas et al., thrombocytopenia in COVID-19 patients has been shown to increase the mortality of patients with severe COVID-19 infection [12]. Identical to SARS-CoV and human corona virus-229 E (HCoV-229E) infections, SARS-CoV-2 infection results in thrombocytopenia. Depending on this occurrence, it has been hypothesized that SARS-CoV-2 exerts a comparable inhibition of hematopoiesis in the bone marrow through specific receptors, resulting in reduced primary platelet production and thrombocytopenia [13].

Various biomarkers linked to multi-organ failure in severe COVID-19 infection include pro-inflammatory cytokines, albumin, Ferritin, bilirubin, alanine transaminase, LDH, urea, creatinine, and eGFR [14]. In the present study, the total leukocyte count, CRP, urea, creatinine, IL-6, and LDH were significantly more significant, and platelet count was significantly lower in the non-survivors group, showing the SARS-CoV-2 infection-associated multi-organ failure. Our findings are similar to a previous study, which reported significantly elevated leukocyte count, neutrophil count, N/L ratio, and decreased platelet count among the non-survivors of COVID-19 infection (4). Similar studies have also reported the same laboratory parameters in patients with severe COVID-19 infection admitted to the ICU [15,16].

It is well known that CRP is a sign of severe infection and systemic inflammation. In this study, CRP was significantly elevated among non-survivors. Previous studies in COVID-19 patients have reported that CRP levels were related to mortality with an area under the receiver operating characteristic curve (AUC) of 0.896. Patients who died had an initial CRP 10 times greater than survivors (100.0 vs. 9.7 mg/L, P 0.001) [17]. In our study, urea, creatinine, and LDH were also significantly elevated among non-survivors; Ferritin and D-dimer were also elevated among non-survivors though statistically non-significant. According to Wang et al., severely ill COVID-19 patients had substantially higher levels of aspartate transaminase (AST), ALT, LDH, and D-dimer. In individuals with COVID-19 infection, Ferritin, along with CRP and IL-6, is linked to catastrophic results [18].

In the present study, the multivariate logistic regression for predictors of mortality showed CT severity score (CT-SS), NIPPV, and IL-6 had a relative risk of 1.17, 0.052, and 1.03, respectively. In our study, the CT severity score was significantly higher among non-survivors. This is similar to previous studies, which reported a substantial difference between individuals with mild to moderate COVID-19 and those who died from it in terms of the CT severity score of lung involvement [19]. Zakariaee et al. investigated the association of CT-SS with the mortality of COVID-19 patients in their meta-analysis, and the final pooled OR was calculated as 1.244 (95% CI 1.157-1.337). The outcome supports the hypothesis that CT-SS and COVID-19 mortality are directly correlated. There are greater mortality probabilities for COVID-19 patients with higher CT severity scores [20].

In the present study, the use of NIPPV was significantly greater among non-survivors. This is consistent with previous studies that reported that the use of NIPPV was greater among hospitalized patients with severe COVID-19 infection [21,22]. IL-6 is an acute-phase inflammatory mediator that rises within 2 to 3 hours of infection and is associated with a poor prognosis in COVID-19 patients, reflecting the intensity, degree of pulmonary inflammation, and infection. It has also been noted that patients with COVID-19 infection and IL-6 levels >240 pg/mL have worse outcomes [23]. Studies have shown that rising levels of IL-6 are correlated with disease severity and are particularly useful in identifying individuals who have progressed to more severe stages of COVID-19 [24]. Our study shows that the inflammatory marker IL-6 proves to be a better indicator of the outcome of COVID-19, with a sensitivity of 81.5% and specificity of 81.8% with a cut-off value of 37.5.

A limitation of our study was that it used a sample size of 125 individuals and was done at a single tertiary healthcare facility. Being a retrospective study, it has its limitation of documentation by the observer.

## Conclusions

The clinical, hematological, and biochemical characteristics of COVID-19 infection play a crucial role in predicting the outcome, which may be taken into account by the physician when planning future decisions. These signs may help clinicians identify cases with a high likelihood of mortality and those with better prognoses during the first admission stage. As a clinical sign of possible critical illness development, we advise monitoring total leucocyte count, CRP, urea, creatinine, IL-6, LDH, platelet count, and CT severity score in COVID-19 patients. IL-6 was found to be a significant marker as a predictor of outcome in our study.

## References

[1] Cascella M, Rajnik M, Aleem A, Dulebohn SC, Di Napoli R (2022). Features, Evaluation, and Treatment of Coronavirus (COVID-19). https://www.ncbi.nlm.nih.gov/books/NBK554776/.

[2] Shi C, Wang L, Ye J (2021). Predictors of mortality in patients with coronavirus disease 2019: a systematic review and meta-analysis. BMC Infect Dis.

[3] Leulseged TW, Hassen IS, Ayele BT (2021). Laboratory biomarkers of COVID-19 disease severity and outcome: findings from a developing country. PLoS One.

[4] Sakthivadivel V, Bohra GK, Maithilikarpagaselvi N (2021). Association of inflammatory markers with COVID-19 outcome among hospitalized patients: experience from a tertiary healthcare center in Western India. Maedica (Bucur).

[5] Welch C (2021). Age and frailty are independently associated with increased COVID-19 mortality and increased care needs in survivors: results of an international multi-centre study. Age Ageing.

[6] Leng J, Goldstein DR (2010). Impact of aging on viral infections. Microbes Infect.

[7] Biswas M, Rahaman S, Biswas TK, Haque Z, Ibrahim B (2020). Association of sex, age, and comorbidities with mortality in COVID-19 patients: a systematic review and meta-analysis. Intervirology.

[8] Fang L, Karakiulakis G, Roth M (2020). Are patients with hypertension and diabetes mellitus at increased risk for COVID-19 infection?. Lancet Respir Med.

[9] Schiffrin EL, Flack JM, Ito S, Muntner P, Webb RC (2020). Hypertension and COVID-19. Am J Hypertens.

[10] Mejía F, Medina C, Cornejo E (2020). Oxygen saturation as a predictor of mortality in hospitalized adult patients with COVID-19 in a public hospital in Lima, Peru. PLoS One.

[11] Xie J, Covassin N, Fan Z (2020). Association between hypoxemia and mortality in patients With COVID-19. Mayo Clin Proc.

[12] Rampotas A, Pavord S (2021). Platelet aggregates, a marker of severe COVID-19 disease. J Clin Pathol.

[13] Xu P, Zhou Q, Xu J (2020). Mechanism of thrombocytopenia in COVID-19 patients. Ann Hematol.

[14] Zemlin AE, Allwood B, Erasmus RT (2022). Prognostic value of biochemical parameters among severe COVID-19 patients admitted to an intensive care unit of a tertiary hospital in South Africa. IJID Reg.

[15] Shang Y, Liu T, Wei Y (2020). Scoring systems for predicting mortality for severe patients with COVID-19. EClinicalMedicine.

[16] Sheng L, Wang X, Tang N, Meng F, Huang L, Li D (2021). Clinical characteristics of moderate and severe cases with COVID-19 in Wuhan, China: a retrospective study. Clin Exp Med.

[17] Liang W, Liang H, Ou L (2020). Development and validation of a clinical risk score to predict the occurrence of critical illness in hospitalized patients with COVID-19. JAMA Intern Med.

[18] Wang D, Li R, Wang J (2020). Correlation analysis between disease severity and clinical and biochemical characteristics of 143 cases of COVID-19 in Wuhan, China: a descriptive study. BMC Infect Dis.

[19] Hu Y, Zhan C, Chen C, Ai T, Xia L (2020). Chest CT findings related to mortality of patients with COVID-19: a retrospective case-series study. PLoS One.

[20] Zakariaee SS, Salmanipour H, Naderi N, Kazemi-Arpanahi H, Shanbehzadeh M (2022). Association of chest CT severity score with mortality of COVID-19 patients: a systematic review and meta-analysis. Clin Transl Imaging.

[21] Grasselli G, Zangrillo A, Zanella A (2020). Baseline characteristics and outcomes of 1591 patients infected with SARS-CoV-2 admitted to ICUs of the Lombardy region, Italy. JAMA.

[22] Guan WJ, Ni ZY, Hu Y (2020). Clinical characteristics of Coronavirus disease 2019 in China. N Engl J Med.

[23] Broman N, Rantasärkkä K, Feuth T (2021). IL-6 and other biomarkers as predictors of severity in COVID-19. Ann Med.

[24] Santa Cruz A, Mendes-Frias A, Oliveira AI (2021). Interleukin-6 is a biomarker for the development of fatal severe acute respiratory syndrome coronavirus 2 pneumonia. Front Immunol.

